# The serotype distribution of *Streptococcus agalactiae* (GBS) carriage isolates among pregnant women having risk factors for early-onset GBS disease: a comparative study with GBS causing invasive infections during the same period in Denmark

**DOI:** 10.1186/s12879-021-06820-2

**Published:** 2021-11-01

**Authors:** Hans-Christian Slotved, Jens Kjølseth Møller, Mohammad Rohi Khalil, Stine Yde Nielsen

**Affiliations:** 1 Department of Bacteria, Parasites and, Fungi, Statens Serum Institut, Artillerivej 5, DK-2300 Copenhagen, Denmark; 2 Department of Clinical Microbiology, Vejle Hospital, University Hospital of Southern Denmark, Vejle, Denmark; 3 Department of Gynecology and Obstetrics, Kolding Hospital, University Hospital of Southern Denmark, Kolding, Denmark; 4 Department of Biomedicine, Aarhus University, Aarhus, Denmark

**Keywords:** Group B streptococci, *Streptococcus agalactiae*, EOGBS, Pregnancy, Carriage, Antimicrobial susceptibility

## Abstract

**Background:**

We describe the serotype distribution of *Streptococcus agalactiae* (GBS) carriage isolates from women in labor and among GBS isolates causing invasive infections during the same period to see if the distribution of carriage serotypes reflects the GBS serotypes causing invasive diseases including early-onset disease (EOGBS).

**Methods:**

Data on invasive isolates from 2019 including serotype, erythromycin and clindamycin susceptibility was retrieved from the Danish national reference laboratory, Statens Serum Institut. Carriage isolates were collected from women with risk factors for EOGBS enrolled at delivery at the maternity ward at a Danish University Hospital, first half of 2019.

**Results:**

Among carriage isolates, the dominant serotype was IX (21 %) followed by serotype III (19 %). The resistance to erythromycin and clindamycin was 21 and 26 %, respectively. Among invasive GBS isolates, no case of EOGBS with serotype IX was detected but the distribution of serotypes were otherwise similar to the GBS carrier strains. The corresponding resistance to erythromycin and clindamycin was 23  and 15 %, respectively. Penicillin resistance was not detected among carriage nor invasive isolates.

**Conclusions:**

The distribution of serotypes among carriage and invasive GBS reflects the assumption that EOGBS occur following transmission of GBS from mother to newborn, with the exception of serotype IX.

**Supplementary Information:**

The online version contains supplementary material available at 10.1186/s12879-021-06820-2.

## Background

*Streptococcus agalactiae* (group B streptococci, GBS) is considered a significant pathogen causing infections in the newborn, the elderly, and adults with underlying medical conditions [1]. In the newborn, invasive infection is generally presented as an early-onset disease (EOGBS) during the first week of life. The mother is considered a source of transmission of the GBS infection [2]. Studies have shown that carriage rates with GBS in pregnant women can vary from 10 to 35 % cultured at gestational week 35–37, although this can only be considered as a qualified guess for the rate at birth since GBS colonization may be transient and vary during pregnancy. [3–6].

Invasive GBS disease occurring from seven days of life and up to three months of age is defined as a late-onset disease (LOGBS) and is generally due to transmission from the infant’s surroundings [2]. The overall incidence of Danish invasive GBS infections in 2019 was 3.53 per 100,000, which was an increase in incidence from 2018 (3.48 per 100,000) and a continuation of the general increase of GBS infections observed in recent years in Denmark [1]. Similarly, the incidence of EOGBS among Danish newborns increased from 2018 (0.15 per 1.000) to 2019 (0.29 per 1.000) [1].

Besides being a human pathogen, GBS is also a commensal of the gastrointestinal tract and vagina. GBS strains isolated from humans are currently divided into ten serotypes based on serotype-specific antigens and are designated as Ia, Ib, II, III, IV, V, VI, VII, VIII, and IX [1]. The serotypes found in carriers are similar to strains causing invasive diseases. In Europe, however, the serotypes Ia, Ib, II, III, and V are the most prevalent carriage isolates [7, 8] while the most prevalent serotype causing invasive disease in Denmark is serotype III followed by serotypes Ia, V, II, and Ib [1].

Two well-known strategies for preventing EOGBS exist. The first strategy is the risk-based approach, where intrapartum antibiotic prophylaxis (IAP) is given to those women with one or more of risk factors for EOGBS [9–11]. The second strategy is based on universal culture screening for GBS carriage at gestational week 35-37 and IAP is administered to all GBS-positive women during labor. This strategy led to a reduction in the EOGBS incidence [9, 11, 12]. However, as a consequence of the universal culture screening at gestational week 35-37, more than 30 % of infants delivered in the United States are currently exposed to intrapartum antibiotics in order to prevent vertical transmission of GBS to the newborn [13].

In Denmark, the risk based strategy is still recommended, although some hospitals now use intrapartum GBS screening using Polymerase Chain Reaction (PCR) when evaluating the risk assessment in order to limit the use of intrapartum antibiotic prophylaxis [14]. The Danish recommendations for screening of GBS is as described [15]:

Intrapartum PCR test for GBS is recommended to the following women: (A) Women given birth with rupture of membranes > 18 timer (The answer of the PCR test will be present before 18 h has passed). (B) Women given birth with a gestational age of 35 + 0 weeks to 36 + 6 weeks. (C) Women who have been treated for GBS urinary tract infection during the current pregnancy.

The following women are also PCR tested for guidance: (A) Women with a temperature >38.0 ˚C during labor. (B) Women in preterm birth before week 35 + 0. (C) Women, who, in a prior pregnancy, gave birth to a child with invasive GBS infection or where GBS infection was suspected.

This study aimed to determine the prevalence and serotype distribution of GBS carriage strains in laboring women with the risk of delivering a newborn with EOGBS and compare these strains to invasive GBS strains causing bloodstream infection in all age groups including EOGBS. We specifically wanted to explore if the serotype distribution among carriage strains reflects the GBS serotypes causing EOGBS. We also compared the antibiotic susceptibility of the carriage isolates with that of the GBS strains isolated from patients with EOGBS.

## Methods

### Study population and sample collection, women in labor

The carriage study was performed at Kolding Hospital, University hospital of Southern Denmark. The hospital represent a population of 300,544, serving as the referral center for woman in labor. Isolates and data on GBS carriers are part of a previous study where details regarding the selection of women can be found [9]. The included women were tested and enrolled prospectively at Kolding Hospital, University hospital of Southern Denmark from December 2018 to July 2019, if they fulfilled at least one of the following criteria for the presence of risk factors for EOGBS: (1) bacteriuria during the current pregnancy, (2) prior infant with EOGBS, (3) temperature above 38.0 °C during labor, (4) preterm labor < 37 gestational weeks, and (5) rupture of membranes ≥ 18 h. Exclusion criteria were women younger than 18 years or women with a communication barrier.

The procedure for rectovaginal sampling was a collection of combined vaginal/rectal swab samples, as described in detail by Nielsen et al. [9].

### Study population, invasive GBS disease

Data on invasive GBS isolates for 2019 were obtained from the Danish laboratory surveillance system at the National Neisseria and Streptococcus Reference Laboratory (NSR), Statens Serum Institut (SSI) as described by Slotved & Hoffmann [1]. Information on age, sex, serotype, origin of the GBS isolates, and date of sampling of the specimens is included in the database. An invasive case was defined as GBS cultured from normally sterile sites such as cerebrospinal fluid or blood. Only one isolate per patient was included in this study, except if different serotypes were isolated from the same patient within 30 days, or if the isolates were detected > 30 days apart [16]. The coverage and evaluation of the database in Denmark are described by Slotved & Hoffmann [1].

For calculation of all incidence data in this manuscript, we obtained population data on both live (per 1000) births and populations (per 100,000) for specific age groups and total population from the Statistics Denmark homepage (https://www.dst.dk/en/Statistik, accessed 19-09-2021).

### Species identification

For details of the laboratory procedure for characterization of GBS isolates from the swab samples, see study by Nielsen et al. [9]. Briefly, samples were examined by PCR test using GenomEra and GeneXpert PCR as well as standard culture of GBS performed on Granada agar plates (BioMérieux) with and without broth pre-enrichment. B-hemolytic orange pigmented colonies from subcultures on Granada agar plates from direct plating as well as from the enrichment broth were identified as *S. agalactiae* by MALDI-TOF (Bruker Daltonik, Germany).

Identification of GBS isolates from submitted invasive samples was performed as described by Lambertsen et al. [17]. The submitted strains were examined for their characteristic beta-hemolytic colonies on 5 % horse blood agar plates (SSI Diagnostica, Denmark) followed by serogrouping with group B latex (Oxoid A/S, Greve, Denmark) as recommended by the manufacturer. Isolates were stored at -80 °C in nutrient beef broth containing 10 % glycerol (SSI Diagnostica, Denmark).

### Serotyping

For identification and serotype procedures see Slotved and Hoffmann [1, 18]. All isolates were serotyped using the GBS latex agglutination test (SSI Diagnostica, Denmark). If the result was inconclusive, the capillary precipitation method (Lancefield method) was applied and this result was considered final. If this procedure did not lead to a phenotypical type designation the isolate was categorized as being non-typable (NT).

### Antibiotic susceptibility testing

GBS isolates from culture-positive carriage samples were tested for antimicrobial susceptibility using disc diffusion. The isolates were screened for sensitivity to erythromycin (15 µg discs), clindamycin (2 µg discs) and penicillin G (1 µg discs). D test was performed to detect inducible clindamycin resistance. Antibiotic susceptibility was determined in accordance with the recommendations by EUCAST (www.eucast.org/clinical_breakpoints).

The invasive isolates were screened using disc diffusion for sensitivity to erythromycin (15 µg discs), clindamycin (2 µg discs) and from 2012 also for sensitivity to penicillin G (1 µg discs). D test was performed to detect inducible clindamycin resistance. For non-susceptible isolates, the minimum inhibitory concentration (MIC) of penicillin, erythromycin, and clindamycin was determined using Etest® (bioMérieux, Denmark).

## Results

### Characteristics of the GBS carriage study group from 2019

A total of 347 women with a median age of 29 years were included in the study. One hundred and one women (median age 30 years) were positive for GBS, while 246 women (median age 29 years) were found negative for GBS. The carriage rate of GBS among pregnant women with risk factors for EOGBS at delivery was 29 %.

No children of the participating women in labor were diagnosed with EOGBS nor LOGBS.

**Fig. 1:**
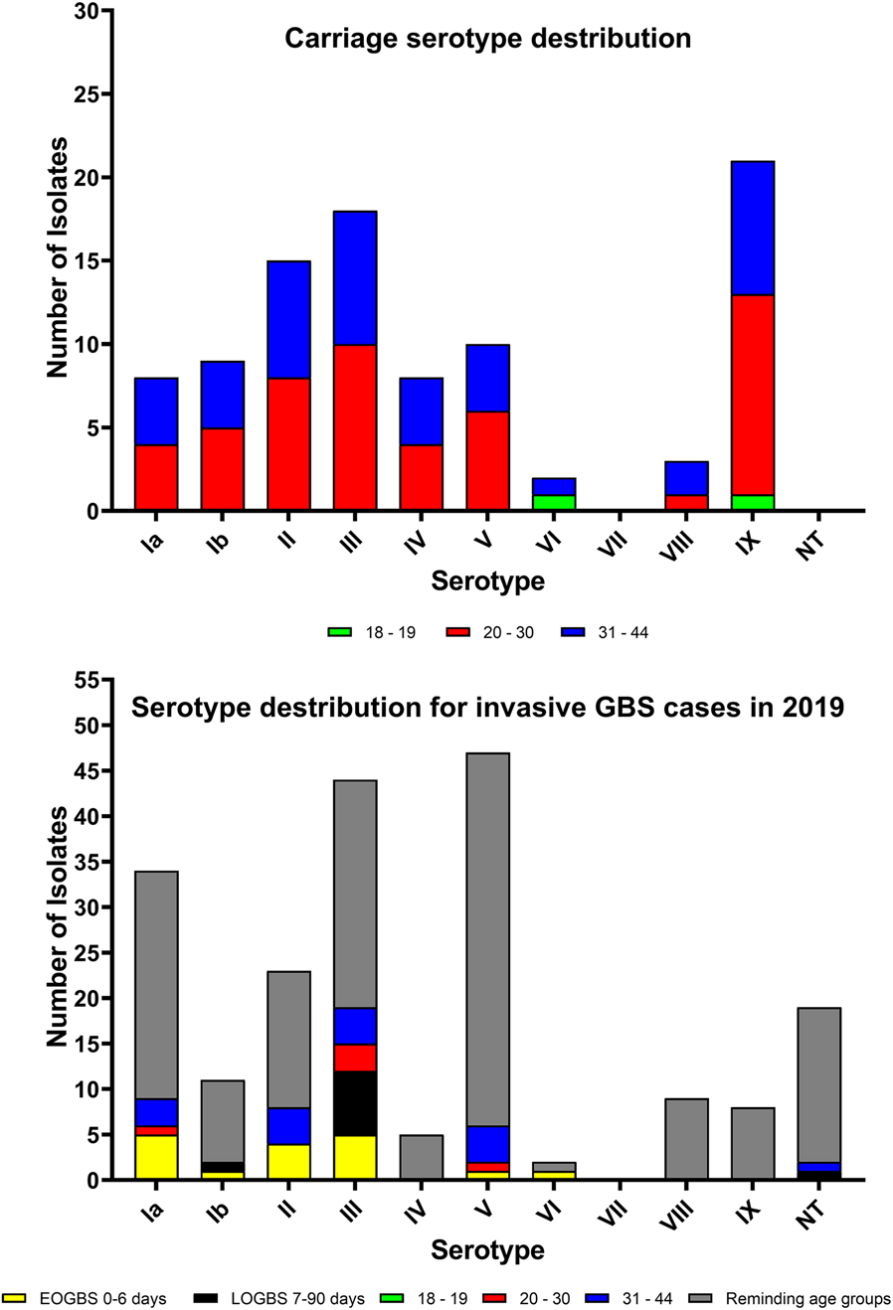
Distribution of serotypes among GBS carrier isolates from woman at risk for EOGBS at birth and invasive GBS isolates from Denmark detected in 2019

All known serotypes were detected except for serotype VII (Table 1; Fig. 1). The predominant serotype was serotype IX (21.0 %), followed by serotype III (18.8 %), and serotype II (14.9 %). No non-typeable isolates were detected.

**Table 1:** Serotype distribution for invasive (from both female and male) and GBS carrier (only from female) isolates

	Invasive cases in Denmark^4^						Carriage in laboring woman			
GBS serotypes	EOGBS [M/F]	LOGBS [M/F]	18 – 19 years [M/F]	20 – 30 years [M/F]	31 – 44 years [M/F]	Total [M/F] (%)	18 – 19 years	20 – 30 years	31 – 44 years	Total (%)
Ia	5 [2/3]	0	0	1 [0/1]	3 [1/2]	34 [18/16] (16.8)	0	4	4	8 (7.9)
Ib	1 [1/0]	1 [1/0]	0	0	0	11 [5/6] (5.4)	0	5	4	9 (8.9)
II	4 [4/0]	0	0	0	4 [0/4]	23 [12/11](11.4)	0	8	7	15 (14.9)
III	5 [3/2]	7 [4/3]	0	3 [1/2]	4 [2/2]	44 [23/21](21.8)	0	10	9	19 (18.8)
IV	0	0	0	0	0	5 [2/3] (2.5)	0	4	4	8 (7.9)
V	1 [0/1]	0	0	1 [0/1]	4 [0/4]	47 [28/19](23.3)	0	6	4	10 (9.9)
VI	1 [1/0]	0	0	0	0	2 [2/0](1.0)	1	0	1	2 (2.0)
VII	0	0	0	0	0	0	0	0	0	0
VIII	0	0	0	0	0	9 [4/5] (4.5)	0	1	2	3 (3.0)
IX	0	0	0	0	0	8 [5/3](4.0)	1	12	8	21 (20.8)
NT	0	1 [1/0]	0	0	1 [0/1]	19 [9/10](9.4)	0	1	0	1 (1.0)
Isolate not available	0	0	0	0	0	0	0	3	2	5 (5.0)
Total number	17 [11/6]	9 [6/3]	0	5 [1/4]	16 [3/13]	202 [108/94]	2	54	45	101

*EOGBS* early-onset disease. *LOGBS* late-onset disease. *M/F* male/female. ^4^For invasive cases a data from both female and males included

For additional characteristics of the women included in the carriage study, see Additional file 1: Tables S1 and S2.

### Invasive cases of GBS from Denmark in 2019

The overall incidence of observed invasive GBS cases (per 100,000) was 3.48 while the incidence for EOGBS cases was 0.28 (per 1,000 newborns) (Table 2). The incidence among women in their childbearing age (18 – 44 years) was 1.67 (per 100.000) compared to the incidence for invasive cases in both genders (18 – 44 years) (1.06 per 100.000).

**Table 2:** Incidence and cases of GBS invasive disease in selected age groups in Denmark in 2019 (N = 202)

EOGBS in Denmark, 2019			
	Newborn population	Invasive GBS cases	Incidence per 1000
Total	61,167	17	0.28 (95% CI: 0.15–0.41)
Female	29,648	6	0.20 (95% CI: 0.04–0.36)
Male	31,519	11	0.35 (95% CI: 0.14–0.55)
LOGBS in Denmark, 2019			
	Newborn population	Invasive GBS cases	Incidence per 1000
Total	61,167	9	0.15 (95% CI: 0.05–0.24)
Female	29,648	3	0.10 (95% CI: − 0.01–0.22)
Male	31,519	6	0.19 (95% CI: 0.04–0.34)
Age group 18–19 years			
	Overall population	Invasive GBS cases	Incidence per 100,000
Total	141,501	0	0
Male	72,392	0	0
Female	69,109	0	0
Age group 20–30 years			
	Overall population	Invasive GBS cases	Incidence per 100,000
Total	857,817	6	0.70 (95% CI: 0.14–1.26)
Male	437,846	2	0.46 (95% CI: –0.18–1.09)
Female	419,971	4	0.95 (95% CI: 0.02–1.89)
Age group 31–44			
	Overall population	Invasive GBS cases	Incidence per 100,000
Total	1,057,289	16	1.51 (95% CI: 0.77–2.25)
Male	533,639	3	0.56 (95% CI: –0.07–1.20)
Female	523,650	13	2.48 (95% CI: 1.13–3.83)
Total incidence, 2019, all age groups			
	Overall population	Invasive GBS cases	Incidence per 100,000
Total	5,806,081	202	3.48 (95% CI: 3.00–3.96)
Female	2,917,008	94	3.22 (95% CI: 2.57–3.87)
Male	2,889,073	108	3.74 (95% CI: 3.03–4.44)

*EOGBS* early-onset disease. *LOGBS* late-onset disease. *CI* Confidence interval

All known serotypes, except for serotype VII, were detected as cause of invasive GBS infection in Denmark in 2019 (Table 1; Fig. 1). The predominant serotype was serotype V (23.5 %), followed by serotype III (21.8 %), Ia (16.8 %), and serotype II (11.4 %). Non-typeable isolates constituted 9.4 %. Similar findings were seen for the 17 EOGBS isolates (Table 1) except for the predominance of serotype V.

### Antimicrobial susceptibility

Among Danish invasive isolates in 2019, 23 % were found resistant to erythromycin, and 15 % to clindamycin. Among women in their childbearing age (18–45 years of age), the number of invasive GBS isolates with resistance to erythromycin and clindamycin were 12 % (2 isolates) and 6 % (1 isolate), respectively (Table 3).

**Table 3:** Antimicrobial susceptibility of GBS carrier and invasive isolates. All GBS isolates examined were found penicillin susceptible. Isolates with inducible clindamycin resistance (D-test) are included

Antimicrobial susceptibility pattern carriage isolates (women in labor) (N = 96^1^)	
Antibiotics	Resistant
Erythromycin	20 (20.8 %) (95 % CI: 12.7–29.0 %)
Clindamycin	25 (26.0 %) (95 % CI: 17.3–34.8 %)
Antimicrobial susceptibility pattern of all 203 invasive isolates in 2019 (males and females)	
Antibiotics	Resistant
Erythromycin	45 (22.2 %) (95 % CI: 16.5–27.9 %)
Clindamycin	30 (14.8 %) (95 % CI: 9.9–19.7 %)
Antimicrobial susceptibility pattern of all EOGBS isolates in 2019 (N = 17) (newborn)	
Antibiotics	Resistant
Erythromycin	3 (17.6 %) (95 % CI: − 0.05–35.8 %)
Clindamycin	1 (5.9 %) (95 % CI: − 5.3–17.1 %)
Antimicrobial susceptibility pattern of all LOGBS isolates in 2019 (N = 9) (infants)	
Antibiotics	Resistant
Erythromycin	6 (66.7 %) (95 % CI: 35.9–97.5 %)
Clindamycin	5 (55.6 %) (95 % CI: 23.1–88.0 %)
Antimicrobial susceptibility pattern of isolates from the age group 18–44 in 2019 (N = 22) (males and females)	
Antibiotics	Resistant
Erythromycin	4 (18.2 %) (95 % CI: 2.0–34.3 %)
Clindamycin	3 (13.6 %) (95 % CI: − 0.7–28.0 %)
Antimicrobial susceptibility pattern of isolates from women in their fertile age (18–44 years) in 2019 (N = 17)	
Antibiotics	Resistant
Erythromycin	2 (11.8 %) (95 % CI: − 3.6–27.1 %)
Clindamycin	1 (5.9 %) (95 % CI: − 5.3–17.1 %)

^1^Five isolates were not available.

In the carriage study (women age group 18–45 years of age) the non-susceptibility of isolates for erythromycin, was 21 % and for clindamycin it was 26 %.

Penicillin resistance was neither detected among carriage nor invasive isolates.

## Discussion

The main findings of the study was the dominance of serotype IX (21 %) followed by serotype III (19 %) among randomly selected carriage isolates from women in labor at a Danish maternity ward, whereas no Danish cases of EOGBS with serotype IX was detected in the same time period. Apart from this, the distribution of invasive GBS serotypes in Denmark aligned with the GBS carriage strains from women in labor.

Carriage studies [6, 9] based on rectovaginal swabs from women with risk factors for EOGBS during delivery, showed a carriage rate of 29 %. This is similar to other Danish as well as international studies [19, 20, 7].

In Europe, it is generally observed that the most prevalent carrier serotypes are Ia, Ib, II, III, and V [7]. In our present study, we found that serotype IX was the dominant serotype followed by serotype III, II, V, Ib, and Ia as observed in other European countries (Table 1). The observed serotypes of carriage strains were also found among GBS causing EOGBS in 2019 (Table 1) and constituted 66 % of the serotypes seen among invasive isolates (62 isolates). Interestingly, the serotype IX commonly seen among GBS carriage strains has only caused one case of EOGBS since 2005 (in 2007) [1]. The finding of serotype IX as a dominant serotype has not previously been described in Europe, but a high carriage rate of serotype IX has been observed in Ghana [21] and Argentina [22].

The choice of prophylactic antibiotic treatment of women during labor is penicillin. But in women reporting penicillin allergy, specifically in case of a type-I allergy, erythromycin and especially clindamycin is an alternative. Due to the importance of erythromycin and clindamycin in the treatment of GBS infections, the susceptibility pattern of GBS isolates for these two drugs is closely monitored in many countries [1]. However, there is no routinely performed susceptibility testing of GBS isolates from pregnant nor laboring women in Denmark.

The susceptibility data for carriage isolates presented in this study shows resistance to erythromycin in 21 % and resistance to clindamycin in 26 % of the GBS examined (Table 3), and are comparable to observation from other countries. Although the number of GBS isolates are limited (Table 3), the susceptibility data from GBS carriage isolates and the invasive isolates in this study are relatively similar (Table 3). A similar observation was found in France, with 36.5 % erythromycin resistance and 24.6 % clindamycin resistance in carriage isolates from mainly pregnant women, while 38.9 % erythromycin resistance and 27.5 % clindamycin resistance was observed in invasive GBS isolates from the same age group (Hays et al., 2016). A carriage study from Eastern Sicily (Italy) showed 40 % erythromycin resistance and 31 % clindamycin resistance [8].

In the light of this, the antimicrobial susceptibility of GBS isolates observed in invasive GBS isolates is also presented in the commensal GBS isolates of the gastrointestinal tract and vagina. It is important to monitor the possible derived effect of developing resistance worldwide, as antibiotic resistance is spread via the commensal flora [23].

A strength of our study is the examination and comparison of susceptibility data from both carriage and invasive GBS isolates in the same time period, while studies in general either focus on carriage or invasive GBS isolates. It is, to our knowledge, the first study in Denmark comparing the GBS serotype distribution from carriage in pregnant women to the distribution of GBS serotypes causing invasive diseases from the same time period.

A weakness of our study is the incomplete submission of invasive GBS isolates to the national reference laboratory (SSI) in Denmark. Submission is voluntary and previous studies have estimated a submission rate of about 58 % of all Danish invasive GBS isolates and the submissions are considered to be a random selection [1, 24]. Furthermore, the low number of EOGBS isolates in the material (17/202) may limit the general conclusions of the study.

## Conclusions

In conclusion, we found that serotype IX dominated as carriage isolate, followed by serotype III. In women in labor having risk factors for EOGBS, however, serotype IX was not found in any Danish cases of EOGBS detected in the same time period. In spite of the prevalence of serotype IX, we found that the serotype distribution of GBS carriage strains reflect the serotypes found to cause EOGBS and other types of invasive GBS infections. This aligns with the description of EOGBS as the transmission of GBS from mother to newborn during labor. The observed prevalence of erythromycin and clindamycin resistance rate appear relatively similar between carriage isolates and invasive isolates and should be further monitored in a Danish setting for administering alternative prophylactic antibiotics to women in labor with penicillin allergy.

## Supplementary Information

**Additional file 1:** **Table S1.** Characteristics of the carrier study population. **Table S2.** Distribution of patterns of GBS test result, carrier study (N = 347)

## Data Availability

The datasets used and/or analysed during the current study are available from the corresponding author on reasonable request.

## Abbreviations

GBS: *Streptococcus agalactiae*
Group B streptococci: *Streptococcus agalactiae*
EOGBS: Early-onset disease
LOGBS: Late-onset disease
IAP: Intrapartum antibiotic prophylaxis
NT: Non-typable
MIC: Minimum inhibitory concentration
CI: Confidence interval

## References

[1] Slotved H-C, Hoffmann S (2020). The epidemiology of invasive group B Streptococcus in Denmark from 2005 to 2018. Front Public Heal.

[2] Bulkowstein S, Ben-Shimol S, Givon-Lavi N, Melamed R, Shany E, Greenberg D (2016). Comparison of early onset sepsis and community-acquired late onset sepsis in infants less than 3 months of age. BMC Pediatr.

[3] Hansen SM, Uldbjerg N, Kilian M, Sørensen UBS (2004). Dynamics of Streptococcus agalactiae colonization in women during and after pregnancy and in their infants. J Clin Microbiol.

[4] Verani JR, Schrag SJ (2010). Group B streptococcal disease in infants: progress in prevention and continued challenges. Clin Perinatol.

[5] Bergeron MG, Ke D, Ménard C, François FJ, Gagnon M, Bernier M (2000). Rapid Detection of Group B Streptococci in Pregnant Women at Delivery. N Engl J Med.

[6] Khalil MR, Uldbjerg N, Thorsen PB, Møller JK (2019). Risk-based approach versus culture-based screening for identification of group B streptococci among women in labor. Int J Gynecol Obstet.

[7] Shabayek S, Spellerberg B (2018). Group B streptococcal colonization, molecular characteristics, and epidemiology. Front Microbiol..

[8] Genovese C, D’Angeli F, Di Salvatore V, Tempera G, Nicolosi D (2020). Streptococcus agalactiae in pregnant women: serotype and antimicrobial susceptibility patterns over five years in Eastern Sicily (Italy). Eur J Clin Microbiol Infect Dis.

[9] Nielsen SY, Møller JK, Khalil MR (2020). A comparison of GenomEra® GBS PCR and GeneXpert ® GBS PCR assays with culture of GBS performed with and without broth pre-enrichment. Eur J Clin Microbiol Infect Dis..

[10] Khalil MR, Uldbjerg N, Thorsen PB, Møller JK (2017). Intrapartum PCR assay versus antepartum culture for assessment of vaginal carriage of group B streptococci in a Danish cohort at birth. PLoS One.

[11] Schrag SJ, Zell ER, Lynfield R, Roome A, Arnold KE, Craig AS (2002). A population-based comparison of strategies to prevent early-onset group B streptococcal disease in neonates. N Engl J Med.

[12] Verani JR, McGee L, Schrag SJ, Division of Bacterial Diseases, National Center for Immunization and Respiratory Diseases C for DC and P (CDC)Division of Bacterial Diseases, National Center for Immunization and Respiratory Diseases C for DC and P (CDC) (2010). Prevention of perinatal group B streptococcal disease–revised guidelines from CDC2010. MMWR..

[13] Nanduri SA, Petit S, Smelser C, Apostol M, Alden NB, Harrison LH (2019). Epidemiology of invasive early-onset and late-onset group b streptococcal disease in the United States, 2006 to 2015: multistate laboratory and population-based surveillance. JAMA Pediatr.

[14] Rosenberg LR, Normann AK, Henriksen B, Fenger-Gron J, Møller JK, Khalil MR (2020). Risk-based screening and intrapartum group B streptococcus polymerase chain reactionresults reduce use of antibiotics during labour. Dan Med J.

[15] Brogaard, Lise Roed; Clausen, Tine Dalsgaard; Droogh, Marjoes; Helmig, Rikke Bek; Henriksen, Tine Brink; Khalil, Mohammed; Lousen, Thea; Ludvigsen, Mette; Møller, Nini; Nielsen, Stine Yde; Sørensen, Nicki Broholm Holst; Svare, Jens Anton; Thim SEB. Gruppe B streptokokker—Early onset disease: Profylakse inklusiv GBS screening intrapartum Gruppe. Dansk Selskab for Obstetrik og Gynækologi. 2019; 1–35. https://doi.org/https://www.dsog.dk/obstetrik. Accessed 28 Sep 2021.

[16] Lambertsen LM, Ingels H, Schønheyder HC, Hoffmann S (2014). Nationwide laboratory-based surveillance of invasive beta-haemolytic streptococci in Denmark from 2005 to 2011. Clin Microbiol Infect.

[17] Lambertsen L, Ekelund K, Skovsted IC, Liboriussen A, Slotved HC (2010). Characterisation of invasive group B streptococci from adults in Denmark 1999 to 2004. Eur J Clin Microbiol Infect Dis.

[18] Slotved H-C, Hoffmann S (2017). Evaluation of procedures for typing of group B Streptococcus: a retrospective study. PeerJ.

[19] Johansen NR, Kjærbye-Thygesen A, Jønsson S, Westh H, Nilas L, Rørbye C (2019). Prevalence and treatment of group B streptococcus colonization based on risk factors versus intrapartum culture screening. Eur J Obstet Gynecol Reprod Biol.

[20] Helmig RB, Gertsen JB (2017). Diagnostic accuracy of polymerase chain reaction for intrapartum detection of group B streptococcus colonization. Acta Obstet Gynecol Scand.

[21] Slotved HC, Dayie NTKD, Banini JAN, Frimodt-Møller N (2017). Carriage and serotype distribution of Streptococcus agalactiae in third trimester pregnancy in southern Ghana. BMC Pregnancy Childbirth.

[22] Bobadilla FJ, Novosak MG, Cortese IJ, Delgado OD, Laczeski ME (2021). Prevalence, serotypes and virulence genes of Streptococcus agalactiae isolated from pregnant women with 35-37 weeks of gestation. BMC Infect Dis.

[23] Knowles SJ, O’Sullivan NP, Meenan AM, Hanniffy R, Robson M (2015). Maternal sepsis incidence, aetiology and outcome for mother and fetus: a prospective study. BJOG.

[24] Ekelund K, Skinhoj P, Madsen J, Konradsen HB (2005). Invasive group A, B, C and G streptococcal infections in Denmark 1999-2002: epidemiological and clinical aspects. Clin Microbiol Infect.

