# Supplementary material for: The serotype distribution of Streptococcus agalactiae (GBS) carriage isolates among pregnant women having risk factors for early-onset GBS disease: a comparative study with GBS causing invasive infections during the same period in Denmark
Source: BMC Infect Dis. 2021 Nov 1;21:1129. doi: 10.1186/s12879-021-06820-2 (PMC8561911; doi:10.1186/s12879-021-06820-2)
Supplement: Supplementary file 1 — Additional file 1: Table S1. Characteristics of the carrier study population. Table S2. Distribution of patterns of GBS test result, carrier study (N = 347) [file 12879_2021_6820_MOESM1_ESM.docx]

**The serotype distribution of *Streptococcus agalactiae* (GBS) carriage isolates among pregnant women having risk factors for early-onset GBS disease: A comparative study with GBS causing invasive infections during the same period in Denmark**

**Hans-Christian Slotved^1*^, Jens Kjølseth Møller^2^, Mohammad Rohi Khalil^3^, Stine Yde Nielsen^2,4^**

1 Department of Bacteria, Parasites and, Fungi, Statens Serum Institut, Artillerivej 5, DK-2300 Copenhagen, Denmark.

2 Department of Clinical Microbiology, Vejle Hospital, University hospital of Southern Denmark.

3 Department of Gynecology and Obstetrics, Kolding Hospital, University hospital of Southern Denmark.

4 Department of Biomedicine, Aarhus University.

*Corresponding author: Hans-Christian Slotved, Ph.D., Senior Scientist, Department of Bacteria, Parasites and Fungi, Statens Serum Institut, Artillerivej 5, DK-2300 Copenhagen, Denmark, Tel: +45 3268 8422, E-mail: hcs@ssi.dk.

**Additional file 1: Tables.**

Additional file 1: Table S1. Characteristics of the carrier study population

| Age female participants (total, N = 347) | |
| --- | --- |
| Median | 29 years |
| Age interquartile range | 26 – 32 years |
| Age range | 19 – 44 years |
| Age female participants (GBS positive, N =101) | |
| Median | 30 years |
| Age interquartile range | 26 – 33 years |
| Age range | 19 – 44 years |
| Age female participants (GBS negative, N = 246) | |
| Median | 29 years |
| Age interquartile range | 26 – 43 years |
| Age range | 20 – 43 years |

Additional file 1: Table S2. Distribution of patterns of GBS test result, carrier study (N = 347).

|  |  | N (%) |
| --- | --- | --- |
| Total vagina/rectum samples | | 347 samples |
| Positive vagina/rectum samples | | 101 samples (29.1) |
| Negative vagina/rectum samples | | 246 (70.9) |
|  |  |  |
| Maternal age group (years) | Colonized (%) | Non-colonized (%) |
| < 20 | 2 (100) | 0 (0) |
| 20 – 30 | 54 (25.6) (95% CI^1^: 19.7 – 31.5) | 157 (74.4) (95% CI: 68.5 – 80.3 |
| > 30 | 45 (33.6) (95% CI: 25.6 – 41.6) | 89 (66.4) (95% CI: 58.4 – 74.4) |
| Total all age groups | 101 (29.1) (95% CI: 24.3 – 33.9) | 246 (70.9) (95% CI: 66.7 - 74.7) |

^1^CI: Confidence interval
